# Norovirus inter-species transmission in an ex vivo model is dependent on virus internalization capacity

**DOI:** 10.1080/22221751.2026.2713851

**Published:** 2026-08-03

**Authors:** Nele Villabruna, Myra Hosmillo, Ian Goodfellow, Niklas Arnberg, Juan Bárcena, Silke Rautenschlein, Benjamin Heidrich, Andreas Beineke, Gisa Gerold

**Affiliations:** a Institute of Biochemistry, Research Centre for Emerging Infections and Zoonoses (RIZ), University of Veterinary Medicine Hannover, Hannover, Germany; b Department of Pathology, University of Veterinary Medicine Hannover, Hannover, Germany; c Division of Virology, Department of Pathology, University of Cambridge, Addenbrooke's Hospital, Cambridge, UK; d Department of Clinical Microbiology, Umeå University, Umeå, Sweden; e Umeå Centre for Microbial Research (UCMR), Umeå, Sweden; f The Laboratory for Molecular Infection Medicine Sweden (MIMS), Science for Life Laboratory (SciLifeLab), Umeå University, Umeå, Sweden; g Centro de Investigación en Sanidad Animal (CISA-INIA/CSIC), Madrid, Spain; h Clinic for Poultry, University of Veterinary Medicine Hannover, Hannover, Germany; i Department of Gastroenterology, Hepatology Infectious Diseases and Endocrinology, Hannover Medical School, Hannover, Germany; j Institute of Virology, Medical University of Innsbruck, Innsbruck, Austria

**Keywords:** Norovirus (NoV), virus-like particles (VLPs), zoonotic potential, precision-cut intestinal slices, ex vivo model, virus entry, porcine, canine

## Abstract

Human noroviruses (HuNoV) are the most common cause of viral gastroenteritis worldwide, causing sporadic cases and outbreaks. Noroviruses that infect wild and domestic animals, including pigs and dogs, are genetically related to human noroviruses, increasing the potential for cross-species transmission. To investigate the potential of noroviruses to cross the species barrier, we used porcine, canine, and avian precision-cut intestinal slices. We show that human norovirus virus-like particles (VLPs) bind to pig and dog intestinal tissue and are taken up with a similar efficiency as the porcine and canine noroviruses, respectively. In contrast, no binding or internalization of human, porcine, or canine noroviruses was detected in chicken tissue. We further showed that human norovirus replicates in pig intestinal tissue. In contrast, while animal noroviruses attached to human intestinal cells, intracellular uptake was limited. This suggests that human-to-animal transmission is more likely than animal-to-human transmission and that viral uptake likely presents a species barrier.

## Introduction

*Norovirus* is a genus of the *Caliciviridae* family and the most common non-bacterial cause of foodborne gastroenteritis worldwide. The human norovirus (HuNoV) single-stranded positive RNA genome is composed of three open reading frames (ORF1-3), of which ORF2 is encoding the major structural protein which makes up the viral capsid [1]. Based on capsid amino acid similarity, noroviruses are categorized into ten genogroups (GI–GX) that are further divided into 48 genotypes [2]. Viruses within GI, GII, GIV, GVIII and GIX are commonly found in humans, with GII.4 variants having caused at least six pandemics in the last 20 years [3,4]. GII.4 variants also cause the majority of outbreaks and sporadic infections [4,5]. Viruses of the other genogroups have been detected in a variety of wild and domestic species and are considered species-specific. These include cattle, sheep, and yaks (GIII), cats and dogs (GIV, GVI and GVII), rodents (GV), bats (GX) and some unclassified genogroups that were found in harbour porpoises and Californian sea lions [2]. Exceptions are the porcine genotypes (GII.11, GII.18, and GII.19) and the canine genotype GIV.2, which cluster in the otherwise human norovirus genogroups GII and GIV, respectively. This close genetic relatedness between animal and human noroviruses raises the question of whether these noroviruses could potentially be transmitted between humans and animals.

The detection of human serum immunoglobulin G (IgG) that recognize canine norovirus virus-like particles (VLPs) suggests that there is some exposure to animal noroviruses [6,7]. However, no animal norovirus has been reported in symptomatic or asymptomatic humans to date. In contrast, more studies have reported antibodies recognizing human norovirus VLPs in mammals, primarily in pigs and dogs, but also in some primates. These findings could not only be explained by cross reactivity with animal noroviruses but likely stem from exposure to human norovirus (reviewed in [8]). In addition, several studies have reported the presence of GI and GII (especially pre-GII.4 Sydney variants) human norovirus RNA in stool samples of wild and domestic animals, most commonly in pigs [9]. These sequences shared a 94–100% nucleotide identity with noroviruses detected in humans. Especially with GII.4 variants, the detection in animals postdated the dominant circulation of these variants in the human population, indicating human-to-animal spillover events. These studies usually do not include human samples, and only two studies showed a direct link that included samples of dogs and their owners from the same time period, demonstrating presence of the same human norovirus in humans and animals [10,11]. Caliciviruses infect a range of non-mammalian species, including reptiles, fishes, amphibians, and avian species (e.g. chickens), but noroviruses seem restricted to mammalian species [12]. Nevertheless, two studies have reported human norovirus RNA in bird droppings [13,14].

Regarding potential barriers of cross-species transmission, human experimental infections and *in vitro* studies have shown that human noroviruses use histo-blood group antigens (HBGAs) as attachment factors [15-20]. HBGAs are displayed on intestinal epithelium and are also secreted in bodily fluids like saliva and milk [21]. A subset of the human population, so-called non-secretors, do not present HBGAs in their intestine and are resistant to infection with some noroviruses like GII.4 [17-19]. HBGAs are broadly found among mammalian species [22-24] and have been described to also be attachment factors for canine and bat noroviruses [25,26]. While HBGAs are necessary for norovirus attachment, they are not sufficient for infection. For example, norovirus can attach to human embryonic kidney 293 T cells (HEK293 T) and human hepatoma cells Huh-7 overexpressing HBGAs, and when these cells are transfected with viral RNA, they support limited genome replication; however, the progeny virus cannot infect these cells [27-29]. This indicates that noroviruses readily bind to tissue, but host restriction might occur downstream.

In addition to HBGAs, bile and some bile salts have been shown to affect some norovirus dynamics in humans with different mechanisms being described for different genogroups and genotypes. For GII.3, bile enhances virus endosomal uptake and endosomal acidification, resulting in increased virus replication [30]. For GII.4 Dijon, bile induced VLP clustering and led to an increase in VLP binding and internalization [31]. Murine norovirus interacts directly with bile salts, which causes an allosteric conformational change that subsequently blocks antibody recognition of the protruding domain [32]. Finally, structural studies have shown that bile salts bind directly to the capsid of GII.1, GII.10, and GII.19, enhancing interaction with HBGAs [33].

To investigate norovirus attachment, uptake and replication across species, we investigated the host barrier in porcine, canine and avian hosts. Pigs and dogs were selected for this study because most evidence points to them as potential reservoirs for human noroviruses. Moreover, they are common farm animals and pets that live in close proximity to people, providing ample opportunity for spillover. Chickens were included as control species, as there is little evidence of human or animal norovirus infection in poultry. To study the potential of human noroviruses to infect various animal species *in vitro*, we used precision-cut intestinal slices (PCIS). Precision-cut tissue slices are more commonly used in toxicological and pharmaceutical research; however, they are a promising model to study host-pathogen interactions [34-38]. To also address the potential of animal noroviruses to infect humans, we tested their attachment and internalization to human intestinal cells.

## Material and methods

### Ethics statement

A study approval from an ethics committee was not required, as no additional animal experiments were conducted. The collection of the human bile was approved under ethics vote No. 10853_BO_K_2023 and followed informed patient consent. Samples from dogs and pigs were collected under the permit number DE 03 201 0043 21 obtained from the Fachbereich Öffentliche Ordnung, Gewerbe- und Veterinärangelegenheiten, Landeshauptstadt Hannover. Chicken samples were collected under the permit number TiHo-T-2022-16. Surveillance data were collected and data on molecular characterization of noroviruses were analysed on the basis of routine national infectious disease surveillance duties by local and state health departments and the Robert Koch Institute as laid out in the German Infection Protection Act. Thus, a review by an ethics committee was not required. In accordance with §13 of the German Infection Protection Act, laboratories are permitted to send patient samples to national reference centers and consultant laboratories for further analysis.

### Cells and viruses

Human duodenal adenocarcinoma cells (HuTu80) were cultured in Dulbecco’s modified Eagle medium (DMEM, Sigma-Aldrich, St. Louis, MO, US), supplemented with 100 U/mL penicillin and 100 µg/mL streptomycin (Gibco, Waltham, MA, US), 20 mM HEPES (Thermo Fisher Scientific, Waltham, MA, US), and 10% fetal bovine serum (FBS; Capricorn Scientific, Ebsdorfergrund, Germany) at 37°C and 5% CO_2_.

Stool samples from norovirus-positive individuals were received from the Robert Koch Institute, where they had been typed as GII.4 Sydney[P16] as described by Niendorf et al. [39]. A 10% stool solution was prepared in phosphate-buffered saline (PBS). The samples were sequentially centrifuged at 4°C for 10 min: at 500×*g* to 5000×*g* in 1000 × g increments. Transmissible gastroenteritis virus (TGEV, PUR46 strain) was kindly provided by Paul Becher and Luis Enjuanes.

### Bile samples and bile salts

A human bile sample was taken from a patient at a routine examination at the Department of Gastroenterology, Hepatology, Infectious Diseases, and Endocrinology, Hannover Medical School, Hannover, Germany. Dog bile samples were retrieved from the gallbladder of the same dogs that were also used for intestinal tissue extraction. Chicken bile samples were taken from animals used for other experiments. Pig bile extract was purchased from Sigma-Aldrich (#B8631). All bile samples were aliquoted and stored at −80°C until use.

Glycochenodeoxycholic acid (GCDCA, #700266P) and taurolithocholic acid sodium salt (TLCA, #700252P) were purchased from Merck and Sigma-Aldrich and dissolved in dimethyl sulfoxide (DMSO) to a 50 mM concentration, aliquoted and stored at −20°C.

### Intestinal tissues

For dogs, intestinal tissue was removed during routine necropsy service at the Department of Pathology (1 h to 4 h postmortem). Dogs with intestinal pathology were excluded. Dogs varied in age, sex and breed. No animals were killed for the purpose of this study, and written consent was given by the owners. For pigs, intestinal tissues were received from healthy 6-months-old animals from an abattoir. Chicken tissue was received from specific-pathogen-free healthy animals (Valo, BioMedia GmbH, Osterholz-Scharmbeck, Germany) used for other experiments (females, aged 6–7 months). To exclude the presence of GII norovirus (including HuNoV and PoNoV), intestinal content was extracted from the intestine of each pig, dog, and chicken individual and tested for the presence of norovirus RNA by RT-qPCR as described in the RT-qPCR section.

### Preparation and culturing of precision-cut intestinal slices (PCIS)

PCIS preparation was based on the protocol by de Graaf et al. [40]. Fresh intestinal tissues were harvested from pigs, dogs, or chickens and put into precooled ROTI®Cell Hanks’ BSS with Magnesium and Calcium (Carl ROTH, Karlsruhe, Germany) on ice. Tissues were cut into 4-cm pieces and flushed several times with cold Krebs-Henseleit (KHB) buffer (NaCl 7.00 g/L (Carl ROTH), KCl 0.37 g/L (Carl ROTH), KH_2_PO_4_ 0.16 g/L (Carl ROTH), CaCl_2_× 2H_2_O 0.37 g/L (Carl ROTH), MgSO_4_×7H_2_O 0.27 g/L (Sigma-Aldrich)). The cleaned tissues were cut into 1-cm cross sections and embedded into 8% (w/v) low-melting agarose (#1744-100 g, GERBU, Heidelberg, Germany). The agarose was prepared by dissolving the agarose in sterile water and mixing it with an equal volume of 2× Roswell Park Memorial Institute medium (RPMI, #11544506, Gibco, Life Technologies, Paisley, UK). The agarose was microwaved until fully dissolved and cooled down to ∼37°C to prevent tissue damage. One-centimetre intestinal sections were embedded in the agarose in a 6-well plate and left to solidify at 4°C for 30 min.

The agarose cores were mounted onto a platform of a Vibratome (Leica VT 1200) and cut into 250 µm slices at a speed of 0.2 mm/s and an amplitude of 3 mm. The first ten slices were discarded. The PCIS were collected in ice-cold KHB buffer and transferred to a 12-well plate in 1 mL complete medium (DMEM/Ham’s F12 (#11320033, Thermo Fisher Scientific)), 2.5% FBS, 10 mg/mL human insulin (#I9278, Sigma-Aldrich), 1.4 µg/mL human hydrocortisone (#H6909, Sigma-Aldrich), 5 µg/mL human transferrin (#T8158, Sigma-Aldrich), 1 µg/mL rat fibronectin (#F0635, Sigma-Aldrich), 100 U/mL penicillin/100 µg/mL streptomycin (Lonza, Basel, Switzerland), 50 µg/mL gentamicin (Gibco, was excluded for dog PCIS), 1:100 protease inhibitor (#P8340, Sigma-Aldrich). The PCIS were incubated at 37 °C in 5% CO_2_ and the medium was exchanged twice after 1 h and again after 2 h.

To identify the intestinal section (duodenum, jejunum, ileum), a piece of intestine was collected in ROTI®Histofix 4% Formaldehyde (#A146.6, Carl ROTH) per individual and used for haematoxylin and eosin staining.

### Testing PCIS viability

PCIS viability was assessed by measuring the adenosine triphosphate (ATP) amount and the total protein content to control for variation in PCIS size as reported previously [40,41]. For each timepoint, five PCIS were harvested in 1 mL sonication solution (70% ethanol and 2 mM EDTA), snap frozen and stored at −80°C until further use. Samples were thawed on ice, one Qiagen bead (stainless steel beads 5 mm, #69989) was added per tube, and samples were homogenized in a Qiagen Tissuelyser II at 24 Hz for 30 sec. The samples were centrifuged at 13,000 × g for 2 min at 4°C and diluted 1:10 in ATP buffer (100 mM Tris HCl buffer with 2 mM EDTA, pH 7.6). ATP content was measured using the ATP Bioluminescence Assay Kit CLS II (#11699695001, Roche Diagnostics, Mannheim, Germany) using an ATP calibration curve according to the manufacturer's instructions. For the total protein measurement, the remaining pellet was dissolved in 200 µL of 5 M NaOH and incubated for 30 min at 37°C. The samples were diluted with 800 µL of Milli Q water and the protein content was measured using the BCA kit (#23227, Pierce™ BCA Protein Assay Kit, Thermo Fisher Scientific). For each PCIS, the ratio between ATP content (pmol) and protein content (µg) was calculated. The effect of 5%, 2.5% and 1% bile or 500 µM bile salts was tested by adding the bile (salts) to PCIS for 6 h and subsequently measuring PCIS viability.

### Testing cell viability

The effect of bile and bile acids on HuTu80 cell viability was assessed by MTT (3-(4,5-dimethylthiazol-2-yl)−2,5-diphenyltetrazolium bromide) assay. Twenty-four hours prior, 20,000 cells were seeded in a 96-well plate. Various concentrations of bile (5%, 2.5%, and 1%) and bile salts (500 µM) were added to cells and incubated for 6 h. The supernatant was removed and 50 µL of 1 mg/mL MTT solution (#M6494, Thermo Fisher Scientific) in DMEM were added to the cells and incubated for 2 h. Once blue crystals were detected, the reagent was exchanged for 50 µL of DMSO. Absorbance was determined at wavelengths of 540 and 630 nm using the spectrophotometer Multiskan Go (Thermo Fisher Scientific).

### Labelling of virus-like particles (VLPs) with Alexa Fluor 488 (AF488)

Virus-like particles have been characterized in previous studies and are listed in Supplementary Table 1. GII.4 Dijon, GII.11, and GIV.2 VLPs were generated using the baculoviral-expression system and GII.4 Sydney and GII.3 VLPs were produced in HEK293T cells [23,26,31,42].

For labelling, Alexa Fluor 488-NHS Ester (#A20000, Thermo Fisher Scientific) was dissolved in DMSO at 10 mg/mL. VLPs were incubated at a 40× molar excess, vortexed and incubated for 1 h at room temperature protected from light. Excess dye was removed by buffer exchange through a 30 kDa-Amicon Filter (Millipore, Burlington MA, USA) at 4200×*g* and eluted in PBS. VLP concentration was calculated by Coomassie gel with a bovine serum albumin (BSA) standard.

### Virus histochemistry and immunohistochemistry

The AF488-labelled VLPs were used to test for their binding onto formalin-fixed, paraffin-embedded (FFPE) PCIS based on the previously published method [23]. PCIS were kept in 4% paraformaldehyde (PFA) for 24 h and were embedded into FFPE blocks and cut into 3-µm slices. To detect VLP attachment the avidin–biotin–peroxidase complex method was used. FFPE slides were deparaffinized 3 × 3 min in ROTICLEAR (Carl ROTH, Karlsruhe, Germany) and subsequently rehydrated through a series of alcohols for three min each. Endogenous peroxidase activity was inhibited with 0.5% H_2_O_2_ (in 85% ethanol) for 30 min. Slides were washed three times with PBS and were blocked with 2% BSA for 30 min. AF488-labelled VLPs were added (500 ng in PBS) and incubated overnight at 4°C. The slides were washed three times with PBS and incubated with a rabbit anti-AF488 antibody (1:100 in PBS with 1% BSA, #A-11094, Thermo Fisher Scientific) for 1 h at RT. The slides were washed three times with PBS and incubated with a biotinylated goat anti-rabbit antibody (1:200 in PBS, #BA-9400, Vector Laboratories, Burlingame, CA, USA) for 45 min at RT. The slides were washed three times with PBS and treated with the avidin–biotin–peroxidase complex (#PK6100, Vectastain ELITE ABC Kit, Vector Laboratories) for 30 min at RT. The slides were washed three times with PBS, and the signal was revealed by incubation with 0.05% 3,3′-diaminobenzidine tetrahydrochloride (DAB, Merck KGaA, Darmstadt, Germany) and 0.03% H_2_O_2_ for 5 min. The slides were washed three times with tab water followed by 30 sec nuclear counterstaining with Mayer’s hemalum (Carl ROTH). Slides were washed three times with tab water, dehydrated through a graded alcohol series for 1 min each, followed by 1 min incubation in acetic acid n-butyl ester and xylol and subsequently mounted.

To define the secretor status of each individual through immunohistochemistry, the same protocol was applied as described before but slides were incubated with biotin-labelled *Ulex europaeus* agglutinin 1 (UEA1 (5 ng/µL), #L8262, Sigma-Aldrich) in PBS with 1% BSA over night at 4°C followed by incubation with the biotin amplification complex as described above.

For the α1,2-fucose blocking, slides were incubated with UEA1 (#L5505, Sigma-Aldrich) at 0.1–10 µM for 2 h at RT.

### Entry assay

The assay was based on the published protocol [31]; 24 h prior, 50,000 cells were seeded per well of an 18-well ibiTreat plate (#81816, ibidi, Gräfelfing, Germany). PCIS or HuTu80 cells were incubated with AF488-labelled VLPs (3 µg) for 1 h at 4°C. Unbound VLPs were washed away and the PCIS or HuTu80 cells were transferred to 37°C for 2 h, allowing bound VLPs to be internalized. Samples were transferred to ice and from then on treated with pre-cooled solutions on ice. Samples were blocked with DMEM/Ham’s F12 with 2% BSA (blocking buffer) for 25 min and stained for non-internalized VLPs with a rabbit anti-AF488 antibody (1:500, #A-11094, Thermo Fisher Scientific) in blocking buffer for 1 h. Samples were washed three times with PBS and incubated with a secondary goat anti-rabbit AF568 (1:1000, #A11011, Invitrogen) antibody in blocking buffer for 1 h. The plasma membrane and nuclei were stained with AF647-conjugated wheat germ agglutinin (WGA, 1:500, #W32466, Thermo Fisher Scientific) and Hoechst 33342 (1:5000, #62249, Thermo Fisher Scientific), respectively. Samples were mounted using ProLong™ Diamond Antifade Mountant (#P36970, Thermo Fisher Scientific) and imaged with a Leica SP5 II laser scanning confocal microscope at a 63× magnification. Image settings were consistent for all samples within one experiment. For quantification of VLP entry, at least 100 AF488-positive events were analysed per individual, VLP, and treatment. The images were analysed using CellProfiler 4.2.6 [43]. VLPs were identified as AF488-positive objects (external and internalized VLPs). The images were masked for AF488-positive objects and overlapping AF568-positive objects (only external VLPs) were identified. The fraction of internalized VLPs was calculated as AF488-positive objects (all VLPs) – AF488 + AF568 (external VLPs) and the percentage of internalized versus all VLPs was calculated.

For the α1,2-fucose blocking, PCIS were incubated with UEA1 (#L5505, Sigma-Aldrich) at 0.1– 100 µM for 2 h at room temperature (RT) before the VLPs (3 µg) were added. To test for the blocking efficiency, biotin-labelled UEA1 (5 ng/µL, #L8262, Sigma-Aldrich) and streptavidin-AF647 (10 ng/µL, #S32357, Thermo Fisher Scientific) were used. PCIS and cells were imaged with a Leica SP5 II laser scanning confocal microscope.

To test the effect of bile or bile salts on VLP binding, VLPs were preincubated with 12.5% bile or 4 mM bile salts for 30 min at 37°C. After addition to the PCIS or HuTu80 cells, the final concentration was 3% for bile and 500 µM for bile salts.

### Infection of PCIS

After cutting, PCIS were put at 37°C for 1 h to adjust to the temperature. Virus (1 × 10^7^ genome copies for TGEV and 1 × 10^8^ genome copies for HuNoV) was added and incubated for 2 h at 37°C with 5% CO_2_. The inoculum was removed, PCIS were washed with prewarmed complete medium and fresh medium was added; supernatant of timepoint 0 h was collected. After 24 h, the supernatant was harvested and stored at −80°C. The PCIS were put in 4% PFA for immunofluorescence.

### RT-qPCR

For RNA extraction, the QIAamp Viral RNA Mini Kit (#52906, Qiagen, Venlo, The Netherlands) was used according to the manufacturer’s instructions. The samples were prepared for reverse transcription-quantitative polymerase chain reaction (RT-qPCR) with the LightCycler Multiplex RNA Virus Master kit (#6754155001, Roche). RNA (5 µL) was prepared in a 20 µL reaction with 4 µL RT-qPCR Reaction mix, 1 µL RT enzyme solution, 200 nM primers and probe. The following primer and probe pairs were used: For HuNoV GII: QNIF2d: ATGTTCAGRTGGATGAGRTTCTCWGA, COG2R: TCGACGCCATCTTCATTCACA, QNIFS (Probe): FAM-AGCACGTGGGAGGGCGATCG-TAMRA [44]. For TGEV (nucleocapsid) TGEV_F: TGCCATGAACAAACCAAC, TGEV_R: GGCACTTTACCATCGAAT, Probe: HEX-TAGCACCACGACTACCAAGC-BHQ1 [45]. The reaction was performed in the LightCycler 96 System (Roche Diagnostics) under the following conditions: For norovirus: reverse transcription at 50°C for 15 min, denaturation at 95°C for 5 min, 40 cycles of amplification with denaturation at 95°C for 15 sec and annealing and extension at 60°C for 35 sec. For TGEV: reverse transcription at 50°C for 30 min, denaturation at 95°C for 15 min, 40 cycles of amplification with denaturation at 94°C for 15 sec and annealing and extension at 60°C for 1 min. Genome copy numbers were calculated per PCIS using RNA standards.

### Immunofluorescent stainings

To stain for viral antigen or cell markers, PCIS were fixed in 4% PFA. PCIS were permeabilized in MeOH for 15 min at −20°C. PCIS were blocked in PBS with 3% BSA for 60 min at RT. The primary antibodies were diluted 1:100 in PBS with 3% BSA and 0.05% Tween and incubated overnight at 4°C. As primary antibodies we used mouse anti-norovirus capsid protein (TV-20, 40 ng/µL, R-Biopharm, Darmstadt, Germany) and mouse anti-coronavirus nucleocapsid protein (20 ng/µL, #MA1-82189, Thermo Fisher Scientific). For cell markers we used mouse anti-sucrase isomaltase (4 ng/µL, #sc-393424, Santa Cruz Animal Health, Dallas, TX), rabbit anti-chromogranin A (3 ng/µL, #AB15160, abcam) and UEA1 (5 ng/µL, #L8262, Sigma-Aldrich). After washing, the secondary antibody (AF488-conjugated goat anti-mouse, 20 ng/µL, #A11001, Invitrogen) was added in PBS with 3% BSA and incubated for 1 h at RT. Nuclei were stained with Hoechst 33342 (#62249, Thermo Fisher Scientific). PCIS were washed three times with PBS and mounted in ProLong Diamond Antifade Mountant (#P36970, Thermo Fisher Scientific). Images were taken with a Leica SP5 II laser scanning confocal microscope at a 63× magnification.

## Results

### Setting up precision-cut-intestinal slices of pig, dog and chicken origin

**Figure 1.**
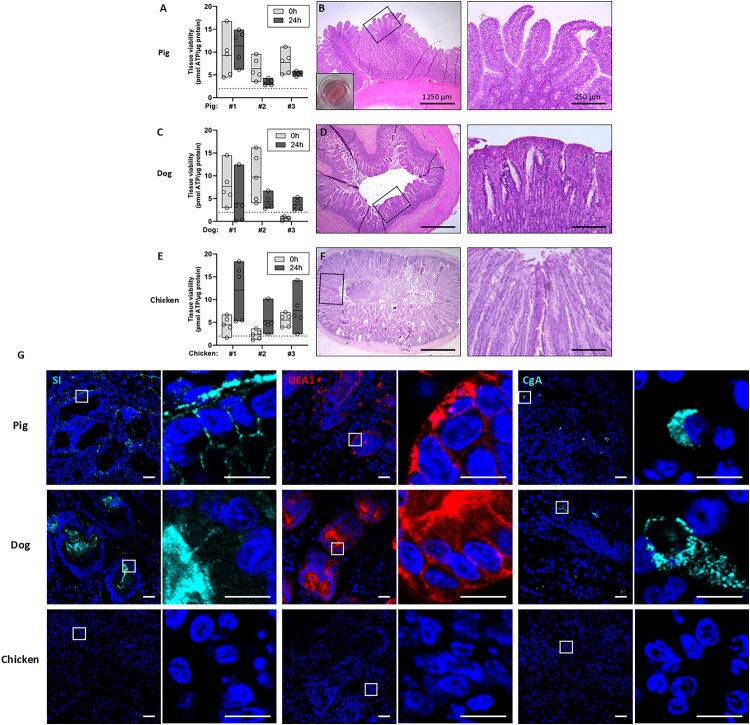
Precision-cut intestinal slices (PCIS) from fresh pig, dog, and chicken small intestinal tissue remain viable for up–24 h. PCIS were prepared by embedding fresh (A and B) pig, (C and D) dog, and (E and F) chicken intestinal tissue in low-melting agarose. The embedded tissues were cut into 250-µm slices and kept in culture medium for 24 h. (A, C, and E). Tissue viability was measured by quantifying the ATP concentration relative to tissue mass (total protein content (µg)). The dotted line indicates the cut-off of 2 pmol ATP/μg protein [40]. Per timepoint and animal, five PCIS were tested. Data of three individuals are shown per species. (B, D, and F) Haematoxylin & eosin (HE) stainings of jejunal tissues of all three species to visualize tissue integrity 24 h after cutting. The black squares indicate the areas that are magnified in the inset. The inset in (B) depicts a PCIS in culture medium. (G) Immunofluorescent stainings of sucrase-isomaltase (SI), *Ulex europaeus* agglutinin 1 (UEA1), and chromogranin A (CgA). Nuclei were stained with Hoechst 33342 (blue). White squares indicate the areas that are magnified in the inset. Scale bars = 25 μm, bars = 10 μm.

To establish PCIS from three animal species, fresh intestinal tissue of pig, dog and chicken origin was obtained and cut as described in the Methods section [46]. PCIS viability was tested at 0 and 24 h post preparation by measuring ATP levels over tissue mass. PCIS viability varied within, but also between species in all three species. The ATP levels in pig and dog PCIS dropped after 24 h to half of the starting concentration, but in chickens they increased to approximately double the starting levels Figure 1 (A,C,E). Despite this drop, the levels were above the 2 pmol ATP/µg protein threshold for tissue viability, as previously defined [40,47]. The macrostructure of the tissue 24 h post cutting consistently showed intact villi and crypts by haematoxylin & eosin and immunofluorescent staining (Figures 1(B,D,F)). In biopsies of immunocompromised individuals, enterocytes and enteroendocrine cells were shown to be target cells for norovirus replication [48,49]. In pig and dog PCIS, we therefore confirmed the presence of these cell types by staining for sucrase-isomaltase (SI for enterocytes) and chromogranin A (CgA for enteroendocrine cells). In addition, the α1,2-fucose group was stained with the (UEA1) lectin (Figure 1(G)). Neither SI-, CgA-, nor α1,2-fucose-positive cells were detected in chicken PCIS, potentially due to a lack of antibody recognition of avian homologues.

### Human norovirus VLPs bind to and are internalized into intestinal tissue of pig and dog origin

HuNoV attaches to the epithelium of formalin-fixed, paraffin-embedded (FFPE) intestinal tissue of a broad range of species [23]. To ensure that the epithelium was preserved after cutting and could still be recognized by norovirus, we tested the binding ability and subsequent internalization of HuNoV GII.4 Dijon, porcine norovirus (PoNoV) GII.11, and canine norovirus (CaNoV) GVI.2 (Supplementary Figure 1) in pig, dog, and chicken intestinal tissues using FFPE PCIS. To that end, we generated virus-like particles (VLP), i.e. particles without encapsidated genome, and labelled them with a fluorophore (AF488).

**Figure 2.**
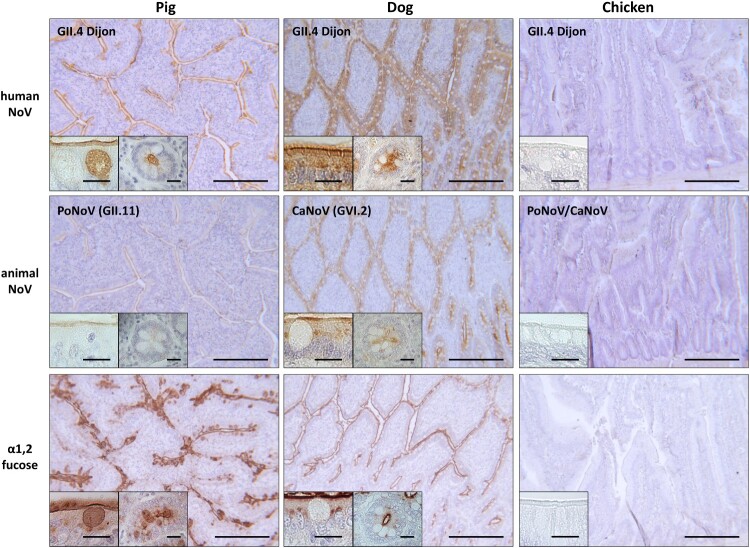
Human norovirus VLPs attach to porcine and canine PCIS, but not to chicken PCIS. Alexa Fluor 488-labelled HuNoV VLPs were tested for their ability to bind to FFPE PCIS. As binding controls, PoNoV (GII.11) and CaNoV (GVI.2) norovirus VLPs were tested on PCIS of their respective hosts. The secretor status of the tissues was confirmed by *Ulex europaeus* agglutinin 1 (UEA1) lectin staining. Brown staining depicts attached VLPs or the presence of a α1,2-fucose group by lectin staining; nuclei are stained in blue (haematoxylin). Insets depict magnifications of goblet cells and crypts. PCIS of seven individuals were tested per species. Images shown are representative. Scale bars = 250 μm, bars = 10 μm.

Bound AF488-labelled VLPs to PCIS of pig, dog, and chicken intestine were visualized by virus histochemistry. HuNoV GII.4 Dijon VLPs attached to the epithelium of all pig and dog PCIS and additionally to some goblet cells in pig but not dog PCIS (Figure 2). As expected, CaNoV VLPs attached to 7/7 dog PCIS, while PoNoV VLPs attached to 2/7 pig PCIS. For the latter, the signal was not as strong as with GII.4 Dijon. Attachment of HuNoV VLPs and CaNoV VLPs was distributed along villi and crypts, while PoNoV VLPs attached only to the villi. Neither the human nor animal norovirus VLPs attached to any of the chicken tissues. Furthermore, all pig and dog, but none of the chicken tissues, tested positive for α1,2-fucose, indicating that all pigs and dogs were secretor-positive and all chickens were secretor-negative.

**Figure 3.**
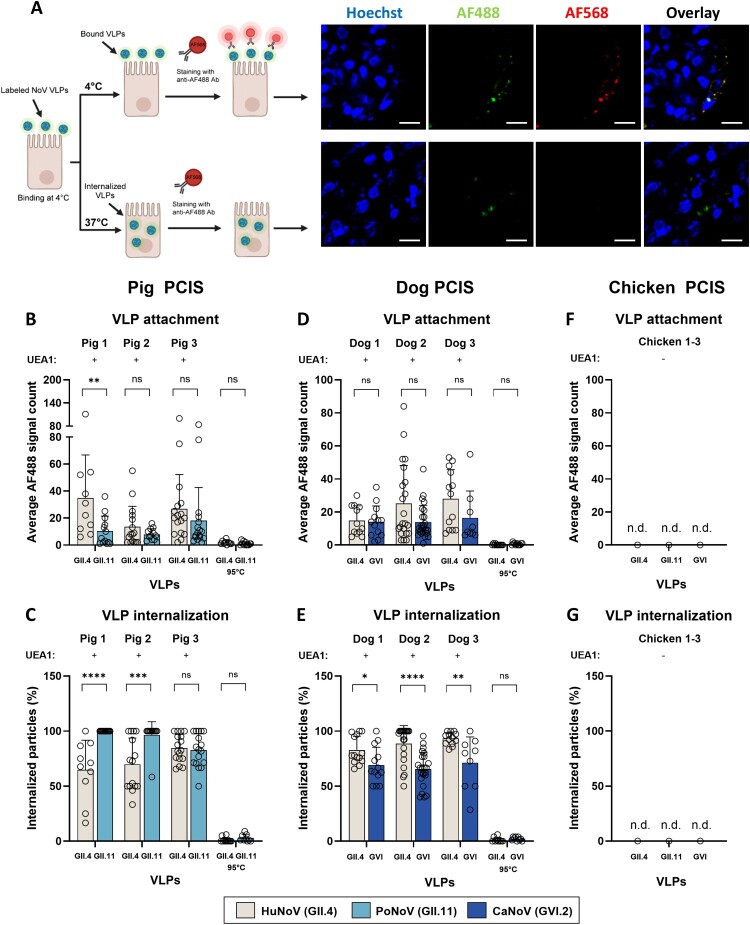
Human norovirus is efficiently taken up into PCIS of pig and dog but not chicken origin. HuNoV VLP binding and internalization was tested in live PCIS. (A, left image) Modified schematic diagram of the entry assay was created in BioRender as described elsewhere [31]. (A, right image) AF488-labelled norovirus VLPs (green) were allowed to bind to animal PCIS on ice (4°C), preventing VLP internalization. An anti-AF488 antibody (AF568-labeled) was added to mark extracellular VLPs, which appear red in addition to the green label. When the temperature was increased to 37°C, VLPs were internalized and no longer accessible to antibodies. Therefore, a single green signal indicates internalized VLPs, while a double green and red signal visualizes extracellular, cell surface-bound VLPs. Nuclei were stained with Hoechst 33342. Representative immunofluorescent images are shown. (B, D, and F) To quantify VLP attachment, AF488 signal per image of the binding control was counted. (C, E, and G). To quantify VLP internalization, the percentage of internalized VLPs (only AF488-positive) versus all VLPs (all AF488-positive events) was calculated. The secretor status of the tissues was confirmed by *Ulex europaeus* agglutinin 1 (UEA1) lectin staining, as indicated on the top of the graph. As control, VLPs were heat-inactivated for 10 min at 95°C. Per species, three individuals were tested. Each data point represents data from one region of interest (ROI). Mann-Whitney test was used to test for significance: **** *P* ≤ 0.0001, ***P ≤ 0.001, **P ≤ 0.1, *P ≤ 0.05, ns = *P* > 0.05. Scale bar =   10 µm in panel A. n.d. =   not detected.

In addition, VLP attachment and internalization were tested in live tissue using an entry assay that allows one to differentiate between attached and internalized VLPs (Figure 3(A)) [31]. HuNoV GII.4 Dijon VLPs attached to and internalized into PCIS of pig and dog origin but not of chicken origin (Figures 3(B–G), Supplementary Figure 2A). PoNoV GII.11 and CaNoV GVI.2 VLPs also attached to and were internalized into pig and dog PCIS, respectively. In pigs, 65–85% of HuNoV VLPs were internalized compared to 83–100% of PoNoV VLPs (Figure 3(C)). In dogs, 89–93% of HuNoV VLPs were internalized compared to 65–71% of CaNoV VLPs (Figure 3(E)). Heat-denaturing VLPs at 95°C for 10 min eliminated binding. Overall, GII.4 Dijon VLPs attached to and were internalized to a similar degree as their respective animal NoV VLPs (Supplementary Figure 2(B)). When comparing the two host species, GII.4 Dijon VLPs attached to and were internalized with similar efficiency to pig and dog PCIS (Supplementary Figure 2(C)).

### Animal noroviruses attach to but are not internalized into human intestinal cells

**Figure 4.**
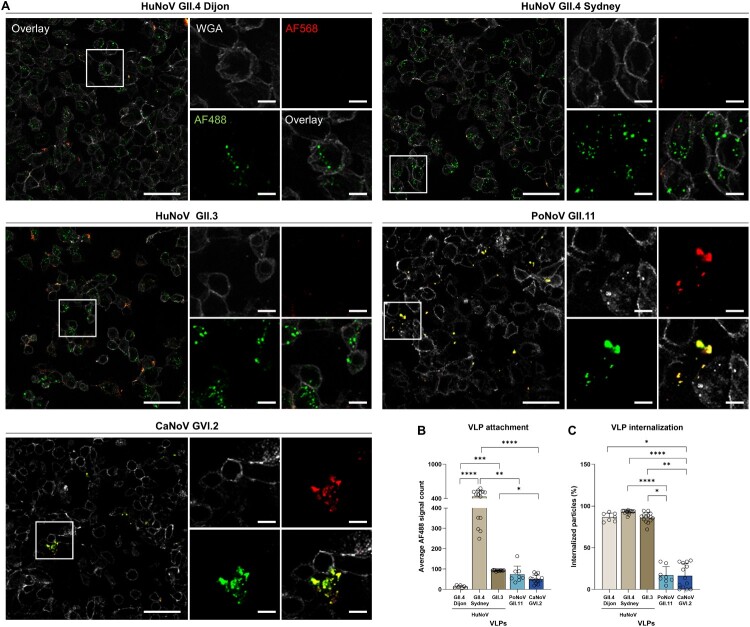
Animal and human norovirus attachment and internalization into human intestinal cells. Human and animal norovirus VLPs attached to and were internalized into HuTu80 cells as described in Figure 3. (A) Immunofluorescent (IF) images of AF488-labelled HuNoV (GII.4 Dijon, GII.4 Sydney, and GII.3), PoNoV (GII.11), and CaNoV (GVI.2) VLPs after 2 h of internalization at 37°C. Membranes were stained with AF647-conjugated wheat germ agglutinin (WGA). (B and C) Quantification of IF images (ROI ≥7). Significant differences in VLP (B) attachment (C) internalization of HuNoV, PoNoV, and CaNoV was tested using One-way Anova, Kruskal-Wallis Test. ****P ≤ 0.0001, ***P ≤ 0.001, **P ≤ 0.  1, *P ≤ 0.05, ns = *P* > 0.05. Scale bar = 50 µm, insets = 10 µm. White squares indicate the magnified areas.

Serological evidence suggests that humans are exposed to PoNoV and CaNoV [6,7,50]. We, therefore, investigated the ability of animal noroviruses to attach to and internalize into human intestinal cells. As no human biopsies were available to produce PCIS, the human duodenal cell line HuTu80 was used instead. Surprisingly, PoNoV and CaNoV VLPs attached significantly better to HuTu80 cells with 74 and 50 average AF488 signal counts, respectively, compared to the HuNoV GII.4 Dijon VLPs with 13 average AF488 signal counts (Figures 4(A,B)). However, only 17% PoNoV VLPs and 16% CaNoV VLPs were internalized, compared to 87% uptake of HuNoV VLPs (Figures 4(A,C)). To test whether this was specific to GII.4 Dijon, we also tested HuNoV GII.4 Sydney and GII.3 VLPs. GII.4 Sydney and GII.3 VLPs attached more efficiently (434 and 93 average AF488 signal counts, respectively) than GII.4 Dijon and the animal norovirus VLPs and were efficiently internalized, at 93% and 86%, respectively.

### HBGA-dependent norovirus attachment to pig and dog PCIS is donor-dependent

**Figure 5.**
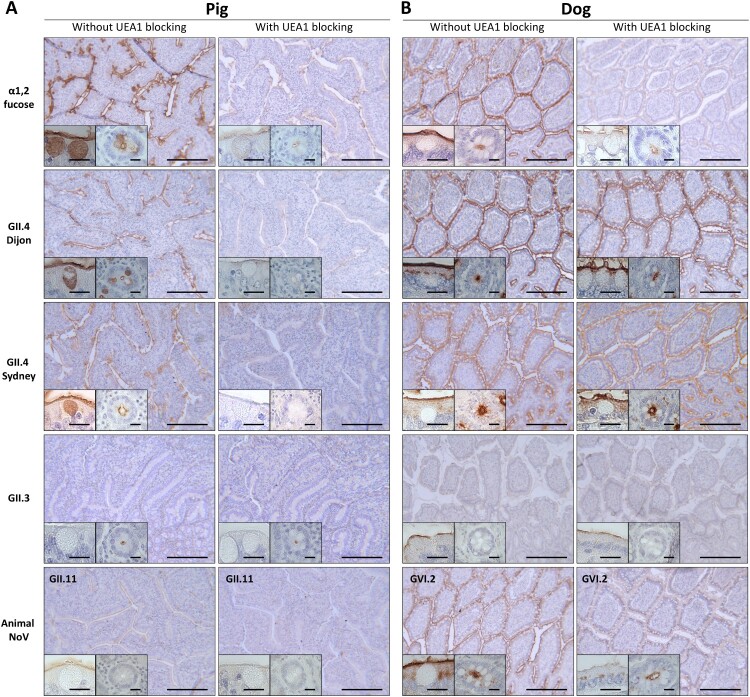
HuNoV VLPs attachment is α1,2-fucose-dependent in FFPE PCIS of pig origin. AF488-labelled norovirus VLPs (500 ng) were tested for their ability to bind to FFPE PCIS of (A) porcine or (B) canine origin. VLP attachment was compared with and without prior blocking of the α1,2-fucose group with 10 µM *Ulex europaeus* agglutinin 1 (UEA1). As binding controls, PoNoV (GII.11) and CaNoV (GVI.2) VLPs were tested on PCIS of their respective hosts. Blocking was confirmed by staining with a biotin-labelled UEA1 (top row). Brown staining depicts attached VLPs or the presence of a α1,2-fucose group by UEA1 lectin staining, nuclei are stained in blue (haematoxylin). Per species, PCIS of three individuals were tested. Results are shown of one individual per species that was representative. Scale bars = 250 µm, insets = 10 µm.

In human organoids, GII.4 infection is strongly dependent on the secretor status [20]. However, blocking the α1,2-fucose group with UEA1 on organoids does not eliminate norovirus VLP binding but influences VLP internalization as well as viral replication [51]. We could previously show that enzymatically removing the α1,2-fucose group eliminated GII.4 Sydney VLP binding and strongly reduced GII.3 VLP binding to fixed human intestinal tissue [23]. To elucidate the role of HBGAs in animal PCIS, we investigated whether blocking the α1,2-fucose group impacts VLP attachment to FFPE and internalization into live PCIS. On FFPE, blocking with 10 µM UEA1 strongly reduced the amount of available α1,2-fucose on the epithelium of porcine and canine PCIS (Figure 5, top row). Upon blocking of the α1,2-fucose group, attachment of GII.4 Dijon, GII.4 Sydney, and PoNoV VLPs (500 ng) to porcine PCIS was strongly reduced, while that of GII.3 VLPs, which only attached to crypt epithelium, was not affected (Figure 5(A)). In contrast, in canine PCIS, only CaNoV VLP attachment was reduced but not that of the GII.4 or GII.3 VLPs (Figure 5(B)). We further tested whether a reduced amount of VLPs could be blocked, but also at 100 ng, VLPs still attached to the crypts in dog tissue (Supplementary Figure 3). This indicates that either the low amounts of available α1,2-fucose suffices for some residual VLP binding or that additional attachment factors, HBGAs or others, play a role.

**Figure 6.**
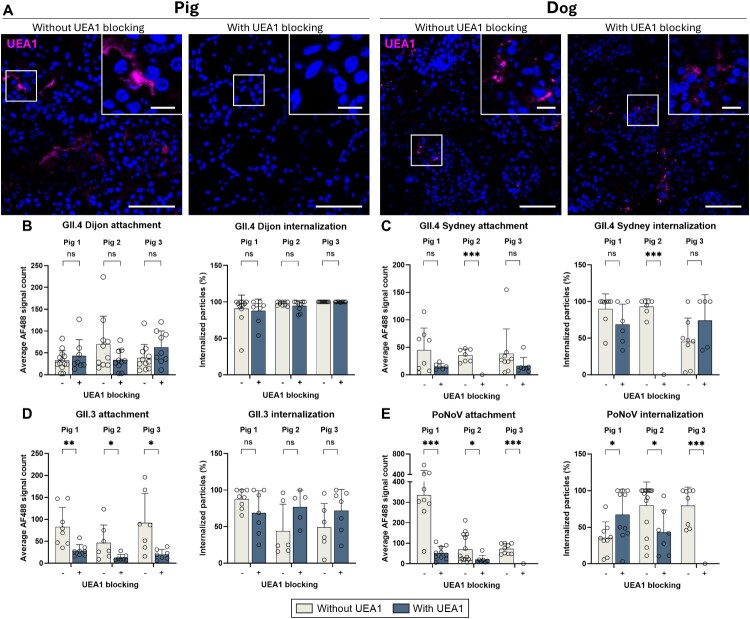
HuNoV VLPs attachment and internalization is not fully α1,2-fucose-dependent in live pig PCIS. (A) Blocking of the α1,2-fucose group with *Ulex europaeus* agglutinin 1 (UEA1) was confirmed in immunofluorescent stainings in pig and dog PCIS. PCIS of pig and dog origin were preincubated with unconjugated UEA1 at 30 and 100 µM, respectively. Blocking was confirmed by staining with biotin-labelled UEA1 and streptavidin-AF647. Nuclei were stained with Hoechst 33342. Representative immunofluorescent images are shown. The effect of UEA1 blocking on pig PCIS of (B) GII.4 Dijon, (C) GII.4 Sydney, (D) GII.3, and (E) PoNoV (GII.11) VLP binding and internalization was tested as described in Figure 3. Per species, three individuals were tested. Each data point represents data from one region of interest (ROI). Mann-Whitney test was used to test for significance: ****P ≤ 0.0001, ***P≤ 0.001, **P ≤ 0.  1, *P ≤ 0.05, ns = *P* > 0.05. Scale bars = 50 µm, insets = 10 µm in panel A. White squares indicate the magnified areas.

To confirm these results in live PCIS, we first confirmed that the α1,2-fucose could be efficiently blocked in non-fixed PCIS. In pig PCIS, 30 µM UEA1 completely blocked the α1,2-fucose group, while in dogs blocking with UEA1 concentrations of up to 100 µM did not efficiently block the α1,2-fucose (Figure 6(A)). The effect of blocking on VLP binding was therefore only tested on pig PCIS. The effect of UEA1 treatment on VLPs attachment and internalization varied between VLPs; GII.4 Dijon attachment and internalization was not significantly reduced in any of the animals tested (Figure 6(B)). GII.4 Sydney attachment was completely blocked in 1/3 individuals (Figure 6(C)). GII.3 VLP attachment was significantly reduced in all three animals, but internalization was unaffected (Figure 6(D)). PoNoV attachment was significantly reduced in all three animals and completely blocked in 1/3 ((Figure 6(E)). PoNoV internalization relative to attached particles was increased in 1/3, decreased in 1/3, and completely blocked in 1/3 animals.

In conclusion, GII.4 and PoNoV VLP attachment is α1,2-fucose-dependent in fixed pig tissues but in live tissues only GII.3 and PoNoV showed this dependency in all individuals. This difference between fixed and live tissues, could indicate that additional attachment factors are available in non-fixed tissues.

### Norovirus attachment is TLCA-dependent in human intestinal cells but not in animal PCIS

In the organoid system, infection of some noroviruses, like GII.3, is dependent on bile, while that of others, including GII.4, increases with bile but is not dependent on it [52]. In HuTu80 cells, human bile and the hydrophobic bile salts glycochenodeoxycholic acid (GCDCA) and taurolithocholic acid (TLCA) increased GII.4 Dijon VLP attachment [31]. We therefore investigated whether bile (salts) also play a role in VLP binding to and internalization into PCIS.

**Figure 7.**
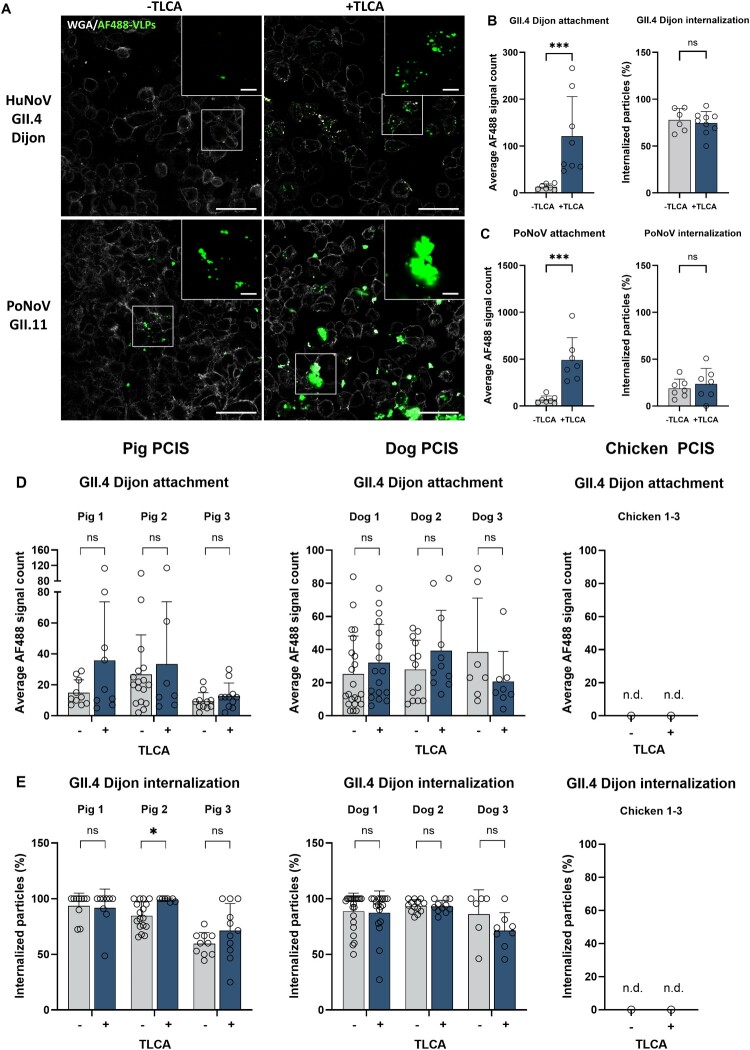
Effect of the bile salt TLCA on GII.4 Dijon VLP attachment to HuTu80 cells and PCIS. VLPs were preincubated with 500 µM TLCA and attachment and internalization measured. (A) Immunofluorescent (IF) images of AF488-labelled GII.4 Dijon and PoNoV (GII.11) VLPs attached to HuTu80 cells at 4°C for 2 h. Membranes were stained with AF647-conjugated wheat germ agglutinin (WGA). Quantification of IF images (ROI ≥7) of (B) GII.4 Dijon and (C) PoNoV (GII.11). (D) GII.4 Dijon VLP attachment to pig, dog, and chicken PCIS. (E) GII.4 Dijon VLP internalization into pig, dog, and chicken PCIS. VLP binding and internalization was tested as described in Figure 3. Per species, PCIS of 3 individuals were included. Each data point represents data from one region of interest (ROI). Mann-Whitney test was used to test for significance: ****P ≤ 0.0001, ***P = ≤ 0.001, ** = *P* ≤ 0.1, * = *P* ≤ 0.05, ns = *P* > 0.05. n.d. = not detected. Scale bar = 50 µm, insets = 10 µm. White squares in A indicate the magnified areas.

We first reproduced the effect of bile and bile salts on GII.4 Dijon VLP binding in HuTu80 cells. As reported by Hahlin et al. [31], preincubation of GII.4 Dijon VLPs with TLCA significantly increased VLP attachment to HuTu80 cells, with no effect on cell toxicity (Figures 7(A,B), Supplementary Figure 4A). This effect was unique to GII.4 Dijon and PoNoV (Figure 7(A–C)), with the other VLPs (GII.4 Sydney, GII.3, and CaNoV) showing no effect or even reduced VLP attachment or internalization upon the addition of bile salts TLCA and GCDCA as well as human or animal bile (Supplementary Figure 4B–E).

To investigate whether TLCA also increased GII.4 Dijon or other HuNoV or animal NoV binding or internalization in animal PCIS, we first tested the effect of the bile and bile salts on PCIS viability. Human and animal bile samples decreased the tissue viability (Supplementary Figure 5A); therefore, only the bile salts were tested. Overall, we did not detect an effect of TLCA on GII.4 Dijon VLP attachment or internalization into animal PCIS (Figures 7(D,E)). We also did not detect an effect of TLCA or GCDCA on attachment or internalization of other VLPs (GII.4 Sydney, GII.3, and CaNoV, Supplementary Figure 5B and C). These data indicate that the effect of bile (salts) on norovirus binding might be specific to human cells.

### Human norovirus replicates in pig PCIS

**Figure 8.**
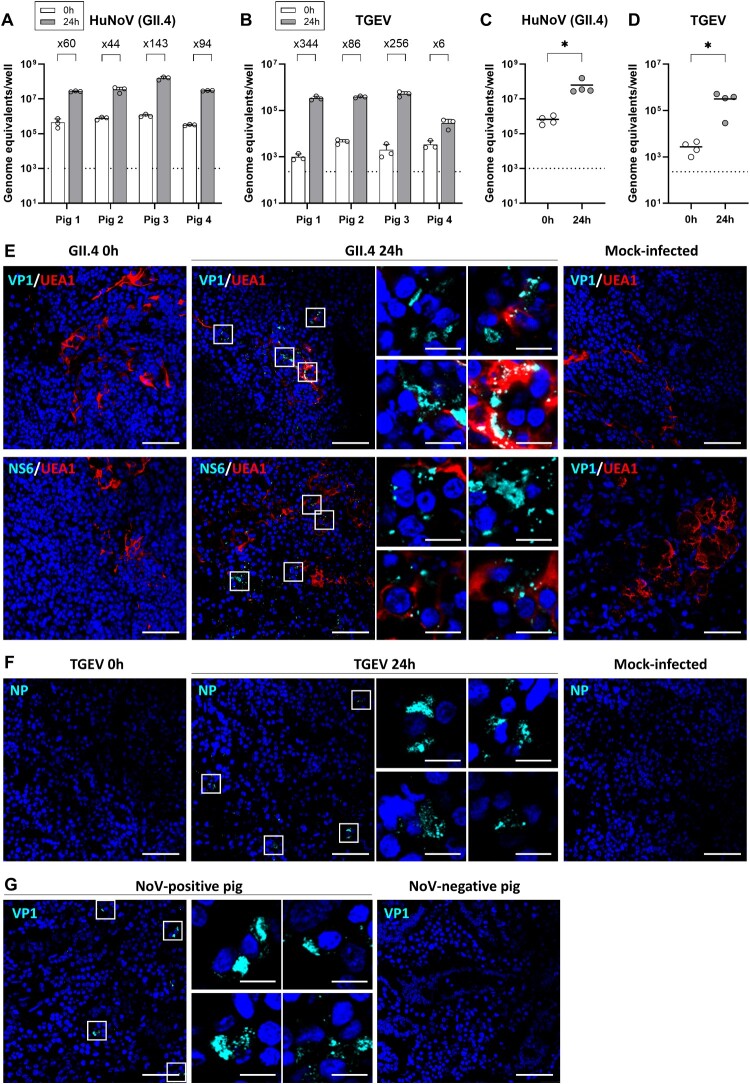
Human norovirus and transmissible gastroenteritis virus (TGEV) replicate in pig PCIS. Pig PCIS were inoculated with (A) HuNoV GII.4 Sydney[P16] or (B) TGEV PUR46 strain and incubated for 1 h at 37°C. Unbound virus was removed, PCIS were washed, fresh medium was added and PCIS were incubated for 24 h at 37°C. The supernatant was harvested right after the washing step for timepoint 0 h and after 24 h. GII.4 and TGEV RNA was detected by RT-qPCR with HuNoV- or TGEV-specific primers and probes. The dotted line indicates the limit of detection. (C and D) Pooled data of 4 animals are shown. For immunofluorescence (IF), HuNoV-inoculated pig PCIS were stained with antibodies recognizing (E) the major capsid protein (VP1) or the norovirus protease (NS6), co-stained with UEA1 lectin staining. (F) TGEV-inoculated pig PCIS were stained with an antibody against the nucleoprotein (NP). (G) IF staining for VP1 of a pig that was RT-qPCR-positive for norovirus and a norovirus-negative pig. Nuclei were stained with Hoechst 33342 (blue). Scale bars = 50 µm, insets = 10 µm. White squares indicate the magnified areas. Mann-Whitney test was used to test for significance: ****P ≤ 0.0001, ***P ≤ 0.001, **P ≤ 0.1, *P ≤ 0.05, ns = *P* > 0.05.

To examine whether internalization of HuNoV into porcine PCIS can lead to virus replication, we inoculated PCIS with GII.4 Sydney[P16] live virus or transmissible gastroenteritis virus (TGEV), a porcine intestinal coronavirus used as a relevant control. After 24 h, we detected an increase in viral genome copy numbers for HuNoV GII.4 (Figures 8(A,C)) and porcine TGEV (Figures 8(B,D)) in PCIS of some animals, with the highest increase of 143-fold and 344-fold, respectively.

By immunofluorescence assay (IFA), using antibodies against the HuNoV structural capsid protein (VP1) and the non-structural protein NS6 (HuNoV protease) or the TGEV nucleoprotein (NP), positive cytoplasmic foci were detected in individual cells, suggesting the presence of HuNoV and TGEV replication in pig PCIS (Figures 8(E,F)). There was no signal at timepoint 0 h or in mock-infected tissues. Stool samples of all pigs used in this study were tested by RT-qPCR for norovirus to exclude natural infection. The stool of one pig was positive for norovirus (5.5 × 10^4^ genome copies/0.1 g stool) and when used for IFA, we also found single VP1-positive cells in this animal (Figure 8(G)). Due to the close genetic similarity between HuNoV and PoNoV, we could not identify the norovirus genotype. Furthermore, we could not propagate the virus from this stool sample in pig PCIS.

## Discussion

Noroviruses infect a broad host range including domestic animals like pigs, dogs, and cows and wild animals like bats, harbour porpoises, and rodents, but the possibility of inter-species transmission remains largely elusive [2]. Here we set out to investigate whether noroviruses can jump the species barrier. We chose pigs and dogs as previous literature points to them as potential reservoirs for human norovirus infection. Chickens served as a control species as there is no link between noroviruses and poultry. We found that HuNoV is not only internalized into intestinal tissue of pig and dog origin but also replicates in pig PCIS between 1.8–2.2 log_10_. In comparison, GII.4 in other norovirus models replicated to 1.5–2.5 log_10_ in organoids, 3.1–3.4 log_10_ in zebrafish larvae, 4.2 log_10_ in zebrafish embryos, and 1–1.4 log_10_ in human B-cell lymphoma (BJAB) cells [52–56].

Virus binding to a host cell is the initial step of the virus life cycle. HuNoV attachment to pig and dog PCIS was comparable to that of host specific PoNoV and CaNoV, respectively. In contrast, none of the VLPs attached to the chicken tissues. These results matched the secretor status as all pig and dog but none of the chicken tissues were secretor positive. The lack of secretor-positive chickens is corroborated by the analysis by Yamamoto *et al*., which found that chickens and other avian species lack the *FUT2* gene [22]. Our findings support results of previous studies using FFPE tissues, where GII.4 attached to a broad range of animal species but not chickens or other avian species, and this correlated with their secretor status [23,26]. As all pig and dog individuals were secretors, we could not investigate the binding pattern to non-secretor individuals.

When we tested for internalization of HuNoV into animal tissue, we found high internalization of >65% in pig and dog tissue, which was comparable to that of the respective animal noroviruses. In a study by White *et al.*, norovirus VLPs were tested for their ability to bind to 10 different mammalian and non-mammalian cell lines [57]. While they bound to all included cell lines, most efficient uptake was seen into differentiated human colorectal adenocarcinoma (Caco-2) cells, into which 19% of VLPs were internalized. In comparison, only 2% were taken up into insect cells. The efficient VLP uptake that we detected suggests that pig and dog intestinal tissue can readily take up norovirus, and that PCIS are a better model to study norovirus entry than Caco-2 cells. In contrast to the comparable uptake rate of human and animal norovirus into animal tissues, we saw a surprising discrepancy in human intestinal cells; although PoNoV and CaNoV attached significantly better to human cells than GII.4 Dijon and less than GII.4 Sydney and GII.3, their uptake was inefficient compared to that of HuNoV (17% vs >85%). This suggests that PoNoV and CaNoV infection of human cells is restricted at the entry step and that human noroviruses are more likely to be transmitted to animals than vice versa, which is supported by a lack of PoNoV or CaNoV sequences in the NoroNet surveillance data from outbreaks or sporadic cases [58]. The standard screening for human noroviruses would detect PoNoV. Screening of human samples for GIV viruses or caliciviruses can furthermore detect CaNoV GIV [59-61].

In a previous study, we could show that GII.4 Sydney VLPs could no longer attach to fixed human intestinal tissue once the α1,2-fucose group was removed [23]. Here, we describe the same effect on fixed pig but not dog tissue, indicating that HuNoV attachment is not necessarily the same between species. This is also supported by the zebrafish model, which lacks the fucosyltransferase 2 and is therefore comparable to the non-secretor phenotype [62]. Zebrafish larvae express lewis x and the α(1,6)-core fucose, the latter of which has not been described as an attachment factor for human noroviruses. In this animal model, α(1,6)-core fucose blocking significantly reduced norovirus infection, indicating that alternative glycan structures could play a role in norovirus infection of non-human species [63]. Furthermore, in non-fixed tissue, blocking of the α1,2-fucose only had an effect in some individuals, indicating that there are also donor-specific differences. Although HBGAs play an important role in host susceptibility for some noroviruses, norovirus interaction with HBGAs alone does not necessarily lead to internalization and virus replication. For example, HBGA-like carbohydrates are also found on bacteria that naturally occur in human stool, and norovirus attaches to these non-eukaryotic cells without infecting them [64]. Similarly, human norovirus attaches to plant cells, especially those from lettuce [65,66]. Attachment presumably occurs via HBGA-like carbohydrates, but it is very unlikely that norovirus particles are taken up, as they were detected exclusively on the outer bacterial membrane and cell wall. With regard to foodborne infections, oysters play an important role in norovirus outbreaks. They express HBGAs similar to humans, thereby bioaccumulating norovirus particles but are not able to sustain infection [67]. Similarly, the human intestinal cell line Caco-2 expresses HBGAs, but efforts to reliably infect these cells have not yielded consistent results [57,68].

When we investigated the effect of bile, we found an effect of TLCA on GII.4 Dijon attachment to human intestinal cells. However, TLCA had no effect on norovirus attachment to PCIS. Unlike in organoid studies, we did not detect an effect of TLCA on GII.3 attachment to PCIS. While we detected an increase in binding and/or internalization in PCIS from single individuals, there was no general pattern. We therefore assume that – as with HBGA – differences between individuals might play a role that is not necessarily considered when working with organoids of one donor. In addition, the GII.3 genotype is diverse, consisting of several clusters, and it is possible that these vary in their response to bile. The effect of bile could also vary between species, as in the zebrafish larvae, GII.3 replicates without the addition of bile [54].

Finally, we could show that human norovirus replicates in pig PCIS, infecting single cells. Not all tissues supported replication, and we do not know yet where this variation stems from. Since all animals were secretor positive, it is likely an HBGA-independent factor. To our knowledge, this is the first study showing that human norovirus can replicate in non-human tissue *ex vivo*.

Working with PCIS offers advantages over cell or organoid cultures, including the preservation of the organ’s spatial organization and underlying pathology. The preparation method is rapid, allowing for experiments to begin within hours after receiving the tissue, and >100 slices can be produced from one donor. Furthermore, similar protocols can be applied to produce PCIS from different mammalian species, and simultaneously, tissue slices from other organs of the same donor can be prepared. Organoid cultures, which are currently used for HuNoV experimentation, are moreover expensive and do not reflect the architecture of the intestine completely.

The most significant limitation of PCIS is their short lifespan of approximately 24 h, making them an inadequate model to study any effect that surpasses this timepoint. In addition, unlike organoids, PCIS cannot be expanded, passaged, or stored for future use. Finally, because PCIS preparation cannot be fully automated, PCIS are unsuitable for high-throughput assays, such as drug screenings.

In conclusion, HBGAs are a crucial factor to initiate norovirus attachment, potentially explaining the lack of binding to chicken intestine. While the presence of HBGAs is necessary for norovirus attachment, it is not sufficient to render a cell line susceptible or even permissive to norovirus infection, as some cell lines that present HBGAs do not support norovirus replication [68,69]. It is likely that HBGA-dependent norovirus attachment does not necessarily initiate virus uptake, indicating the presence of additional susceptibility factors. The nature of co-factor(s) is currently unknown, but an additional protein receptor has been hypothesized to play a role. The high uptake of human norovirus into animal PCIS and the relatively low uptake of animal norovirus into human cells suggests that host restriction occurs between virus attachment and internalization. This is also supported by the lack of norovirus replication in HuTu80 cells, despite the efficient norovirus VLP uptake (Supplementary Figure 6) [70,71]. Hence, norovirus entry should be further investigated. Furthermore, follow-up studies should investigate whether other non-GII.4 GII or GI noroviruses can replicate in pig PCIS, if norovirus acquires host-specific mutations upon passaging, and which other non-α1,2 fucose attachment factors are implicated in norovirus attachment to pig PCIS. Taken together, norovirus transmission from human-to-animal seems more likely than animal-to-human. Whether human noroviruses can be transmitted within an animal reservoir and eventually be transmitted back to humans should be further investigated.

## Supplementary Material

Manuscript Supplementary information without track changes.docx
