# Supplementary material for: Norovirus inter-species transmission in an ex vivo model is dependent on virus internalization capacity
Source: Emerg Microbes Infect. 2026 Aug 3;15(1):2713851. doi: 10.1080/22221751.2026.2713851 (PMC13482930; doi:10.1080/22221751.2026.2713851)
Supplement: Supplementary file 1 [file TEMI_A_2713851_SM2241.docx]

**Supplementary Figure 1. Maximum likelihood tree based on the whole VP1 sequence.** Depicted are norovirus genogroups and genotypes infecting humans, pigs, and carnivores. Porcine and canine/feline noroviruses group within the same genogroups as human noroviruses, GII and GIV, respectively. The genotypes used for VLP binding in this study are marked with arrows.

**Supplementary** **Figure 2. Human NoV VLPs bind to and are taken up into animal PCIS as efficiently as animal NoV.** VLP binding and internalization was tested in an entry assay using live PCIS. (**A**) VLP internalization was quantified by counting AF488-positive events (AF488 – AF568 events). Each data point represents data from one region of interest (ROI). Mann-Whitney test was used to test for significance: **** = P ≤ 0.0001, *** = ≤ 0.001, ** = P ≤ 0.1, * = P ≤ 0.05, ns = P > 0.05. (**B**) HuNoV (GII.4 Dijon) VLPs bind to and are internalized with similar efficiency into pig and dog PCIS as the respective animal NoV, PoNoV (GII.11) and CaNoV (GVI.2). (**C**) GII.4 Dijon VLPs attached to and were internalized with comparable efficiency into pig and dog PCIS. Shown are data of PCIS from seven pigs and four dogs. Significance in panel B was tested using the Wilcoxon matched-pairs signed rank test and in panel C using the Mann-Whitney test was used to test for significance.

**Supplementary Figure 3. HuNoV VLPs attachment is not α1,2-fucose-dependent in FFPE PCIS of dog origin.** AF488-labelled norovirus VLPs (100 ng) were tested for their ability to bind to FFPE PCIS of canine origin. VLPs attachment was compared with and without prior blocking of the α1,2-fucose group with 10 µM *Ulex europaeus* agglutinin 1 (UEA1). Brown staining depicts attached VLPs, nuclei are stained in blue (haematoxylin). PCIS of three individuals were tested. Results are shown of one individual that was representative. Scale bars = 250 µm, insets = 10 µm.

**Supplementary Figure 4. Effect of bile and bile salts on VLP attachment and internalization to HuTu80 cells.** (**A**) Toxicity of 5% bile (human, pig, dog) or 500 µM bile salts (TLCA and GCDCA) on HuTu80 over 6 hours was tested by MTT. Lines indicate 100% and 80% cell viability. (**B** and **D**) The effect of 5% human or pig bile on the attachment and internalization of human and animal norovirus VLPs. (**C** and **E**) The effect of 500 µM bile salts (TLCA or GCDCA) on the attachment and internalization of human and animal norovirus VLPs. Statistical significance was tested between the treatment (bile or bile salts) and the non-treated cells with a Kruskal Wallis test. **** = P ≤ 0.0001, *** = ≤ 0.001, ** = P ≤ 0.1, * = P ≤ 0.05, ns = P > 0.05. NA = not assessed. The dotted line indicates the control.

**A**

**B**

**C**

**Supplementary Figure 5. Effect of the bile salt TLCA on VLP attachment and internalization to animal PCIS.** (**A**) PCIS viability was tested after inoculation with 5% human or animal (pig, dog and chicken) bile samples or bile salts (TLCA and GCDCA, 500 µM) for 6 hours. Tissue viability was tested as before (ATP versus protein content) and the difference is indicated in percentage to negative Ctrl (DMEM/F12 medium for bile samples and DMSO for bile salts). Per species the results of three individuals are shown. The dotted line indicates 100% and 80% viability compared to the control. (**B**) The effect of 500 µM TLCA (30 min preincubation with VLPs at 37°C) on HuNoV (GII.4 Sydney and GII.3) and animal PoNoV (GII.11) and CaNoV (GIV.2) norovirus VLP attachment and (**C**) internalization to animal PCIS. The effect was tested in three animals per species. Mann-Whitney test was used to test for significance: **** = P ≤ 0.0001, *** = ≤ 0.001, ** = P ≤ 0.1, * = P ≤ 0.05, ns = P > 0.05.

**Supplementary Figure 6. Lack of human norovirus replication in HuTu80 cells.** HuTu80 cells (20’000) were seeded in a 96 well plate 24 h prior to infection. Medium was exchanged and cells were inoculated with 1 × 10^8^ genome copy numbers of HuNoV GII.4 Sydney[P16] from six different stool samples (#1 - #6) and incubated for 1 h at 37°C. Unbound virus was removed, cells were washed, fresh medium was added, and cells were incubated for 24 h at 37°C. Cells were harvested after washing step for timepoint 0 h and after 24 h. Per timepoint, three wells were pooled (cells and supernatant) and used for RNA extraction using the QiAmp Viral RNA mini kit 250. HuNoV RNA was detected by RT-qPCR with HuNoV-specific primers and probes.

| **Supplementary Table 1: Virus-like particles used in study** | | | | |
| --- | --- | --- | --- | --- |
| **Genotype**  (NCBI Accession nr) | **Host** | **Binding to HBGAs** | | **Ref** |
|  |  | Synthetic glycans | Saliva |  |
| GII.4 Dijon (=US95_96)  AF472623.1 | Human | H type 1, A heptasaccharide, Lewis b, Lewis y, Lewis x, SiLe x, Si-LNT [1] | O Le-, O Le+, A Le-, Ale+, B Le+ [1] | [2] |
| GII.4 Sydney  (MT232050) | Human | H type 1, H type 3, A, B Lewis b, Lewis y [3] | H type 1, B type, Lewis b, Lewis y [4] | [3] |
| GII.3  (LN854569) | Human | None known [3] | None known [5] | [3] |
| GII.11 (VA34)  AY077644 | Pig | None known [6,7] | None known [6] | [8] |
| GVI.2 (C33)  GQ443611.1 | Dog | H type 1 (HSA),  A heptasaccharide (HSA),  Lewis b (HSA),  Sialyl LNF V (HSA) [9] | O Le-, A Le-, O Le+, A Le+ [9] | [9] |

**References**

[1]	Rougemont Ad, Ruvoen-Clouet N, Simon B, et al. Qualitative and quantitative analysis of the binding of GII.4 norovirus variants onto human blood group antigens. J Virol. 2011;85(9):4057–4070.

[2]	Hahlin E, Danskog K, Nord S, et al. Bile acids accumulate norovirus-like particles and enhance binding to and entry into human enteric epithelial cells. J Virol. 2026;0(0):e00342–26.

[3]	Villabruna N, Schapendonk CM, Aron GI, et al. Human noroviruses attach to intestinal tissues of a broad range of animal species. J Virol. 2020;95(3):e01492–20.

[4]	Morozov V, Hanisch FG, Wegner KM, et al. Pandemic GII.4 Sydney and epidemic GII.17 Kawasaki308 noroviruses display distinct specificities for histo-blood group antigens leading to different transmission vector dynamics in Pacific oysters. Front Microbiol. 2018;9:2826.

[5]	Ayouni S, Estienney M, Sdiri-Loulizi K, et al. Relationship between GII.3 norovirus infections and blood group antigens in young children in Tunisia. Clin Microbiol Infect. 2015;21(9):874.e1–8.

[6]	Yang Y, Xia M, Wang L, et al. Structural basis of host ligand specificity change of GII porcine noroviruses from their closely related GII human noroviruses. Emerg Microbes Infect. 2019;8(1):1642–1657.

[7]	Farkas T, Nakajima S, Sugieda M, et al. Seroprevalence of noroviruses in swine. J Clin Microbiol. 2005;43(2):657–661.

[8]	Almanza H, Cubillos C, Angulo I, et al. Self-assembly of the recombinant capsid protein of a swine norovirus into virus-like particles and evaluation of monoclonal antibodies cross-reactive with a human strain from genogroup II. J Clin Microbiol. 2008;46(12):3971–3979.

[9]	Caddy S, Breiman A, le Pendu J, et al. Genogroup IV and VI canine noroviruses interact with histo-blood group antigens. J Virol. 2014;88(18):10377–10391.
